# Curcumin Modulation of the Gut–Brain Axis for Neuroinflammation and Metabolic Disorders Prevention and Treatment

**DOI:** 10.3390/nu17091430

**Published:** 2025-04-24

**Authors:** Miriam Cerullo, Federica Armeli, Beatrice Mengoni, Martina Menin, Maria Luisa Crudeli, Rita Businaro

**Affiliations:** 1 Neurofarba Department, University of Florence, 50139 Florence, Italy; miriam.cerullo@unifi.it; 2 Department of Medico-Surgical Sciences and Biotechnologies, Sapienza University of Rome, 04100 Latina, Italy; federica.armeli@uniroma1.it (F.A.); beatrice.mengoni@uniroma1.it (B.M.); menin_martina@yahoo.it (M.M.); marialuisa.crudeli@uniroma1.it (M.L.C.)

**Keywords:** curcumin, gut–brain axis, microbiota, systemic inflammation, oxidative stress, metabolic diseases, obesity, BMI, neuroinflammation

## Abstract

Curcumin, a polyphenolic compound derived from Curcuma longa, has gained significant attention for its potential therapeutic benefits, particularly counteracting inflammation, oxidative stress, and metabolic disorders. Its chemical structure, featuring conjugated double bonds between two aromatic rings, allows it to act as an electron donor, thereby mitigating free radical formation. Despite its poor solubility in water, curcumin is stable in acidic environments and undergoes significant metabolism in both the liver and the gut. Intestinal microbiota, particularly at the colon level, further metabolizes curcumin into several derivatives, including dihydrocurcumin and tetrahydrocurcumin, which exhibit antioxidant and anti-inflammatory properties. Studies suggest that curcumin can reduce body mass index (BMI) and improve other body composition parameters, especially when used in combination with lifestyle changes, though its bioavailability is low due to its rapid metabolism and the resulting low blood concentration. In obesity, dysfunctional adipose tissue remodeling and chronic inflammation play critical roles in the development of metabolic complications. Curcumin’s anti-inflammatory properties are related to the inhibition of the NF-κB pathway, leading to the reduction in inflammatory markers in adipocytes and macrophages. Additionally, curcumin modulates oxidative stress by activating the NRF2 pathway, enhancing cellular antioxidant defenses. Emerging evidence also supports curcumin’s potential in improving gut health by modulating microbiota composition, enhancing intestinal barrier function, and reducing systemic inflammation. This interaction with the gut–brain axis highlights the broader implications of curcumin in neuroprotection, as it positively affects cognitive function and mitigates neuroinflammation in neurodegenerative diseases like Alzheimer’s. disease. Thus, curcumin holds promise as a multifaceted agent in the management of obesity and associated diseases.

## 1. Introduction

### 1.1. Polyphenols and Curcumin: Structure and Function

Curcumin belongs to polyphenols, a class of chemical compounds found in plants with phenolic structures, characterized by at least two phenyl rings linked to hydroxyl groups. These compounds, which impart color to many fruits, are crucial for interaction with fruiting animals, facilitating seed dispersal. Inflammation and oxidative stress are the main pathways involved in the development of several chronic degenerative diseases. As a result, there has been growing interest in the role of probiotics and polyphenols as strategies to counteract inflammation and oxidative stress [1,2]. Polyphenols perform several functions in plants, including protecting against oxidative damage, caused by sunlight and other environmental factors, and preserving the organoleptic qualities of plant food products such as olive oil, red wine, and wheat extracts [1,3]. Their chemical structure can vary, from simple compounds to complex polymers with high molecular weight. Polyphenols fall into two groups: flavonoids, which are molecules with two phenolic rings joined by a pyran ring, and non-flavonoids, which possess simpler structures but share at least one common aromatic ring with phenolic acids, lignins, and stilbenes [4]. Curcumin, [1,7-bis(4-hydroxy-3-methoxyphenyl)-1,6-heptadiene-3,5-dione], is the main polyphenolic component extracted from the rhizome of Curcuma longa, a plant in the Zingerberaceae family. Chemically, curcumin has two aromatic rings with conjugated double bonds, which allow it to act as electron donors, preventing the formation of free radicals. Although it is nearly insoluble in water, it dissolves readily in organic solvents such as acetone and ethanol and is stable in acidic environments, such as that of the stomach [5]. Curcumin is resistant to high concentrations of hydrogen ions, which allows it to reach the gut intact after being ingested. Here, it undergoes metabolic processes similar to those that occur in the liver.

### 1.2. Metabolism of Curcumin

Gut and liver enzymes degrade curcumin through phase I and II reactions. In phase I, the molecule undergoes a reduction of double bonds, producing metabolites such as dihydrocurcumin, tetrahydrocurcumin, hexa-hydrocurcumin, and octahydrocurcumin. In phase II, curcumin and its metabolites are conjugated with glucuronic acid and sulfate, mainly at the phenolic site. Glucuronidation is the predominant conjugation pathway, with glucuronide being the major metabolite present in body fluids, organs, and cells [5,6]. In addition to hepatic and intestinal metabolism, recent evidence shows that curcumin is intensively metabolized by bacteria in the intestinal microbiota, particularly in the colon. For example, *Escherichia coli* reduces curcumin to dihydrocurcumin and then to tetrahydrocurcumin, while *Blautia* sp. converts it to bis-demethylcurcumin and demethylcurcumin by demethylation. Other bacteria, such as *Escherichia fergusonii*, *Bifidobacteria longum*, *Lactobacillus*, and others, are also involved in the chemical modifications of curcumin [7].

### 1.3. Curcumin Metabolites and Their Effects

**Figure 1:**
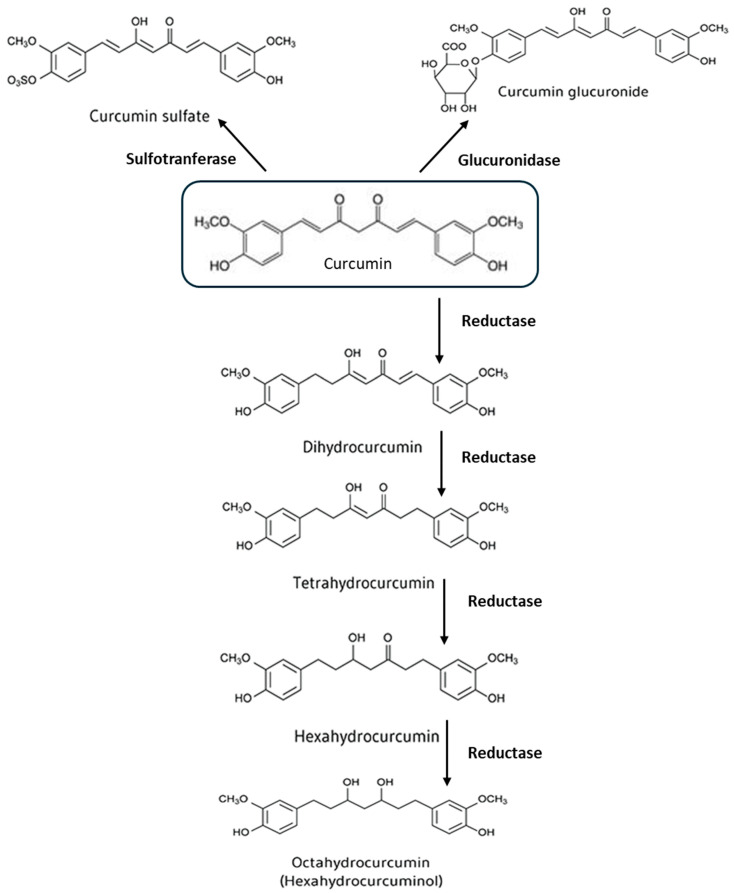
Metabolism of curcumin. Curcumin metabolism occurs mainly through reduction reactions, which generate metabolites such as dihydrocurcumin and tetrahydrocurcumin, which maintain (or sometimes enhance) the biological activity of the original molecule. These metabolites play an essential role in determining the systemic effects of curcumin, despite its low bioavailability.

A recent study showed that the addition of curcumin (100 µM) to bacterial cultures, derived from human fecal samples, generated 23 different types of metabolites, resulting from reactions such as acetylation, demethylation, hydroxylation, and reduction, or combinations thereof. However, the main metabolites were those resulting from reduction reactions (Figure 1) [8]. Some of the metabolites of curcumin showed anti-inflammatory and antioxidant properties similar to those of curcumin itself. For example, tetrahydrocurcumin may prevent oxidative stress and brain inflammation, as well as having potential anticancer effects [5]. Likewise, metabolites such as di-*O*-demethylcurcumin have shown significant neuroprotective properties and are currently being analyzed for the treatment of AD [9]. In general, the effects of the metabolites are still poorly understood, and at present, it is believed that intestinal metabolism, hepatic metabolism, and metabolism carried out by the bacteria are among the main factors causing a decrease in free curcumin levels in the blood [5,6].

### 1.4. Curcumin’s Health Benefits and Applications

Curcumin is widely used, particularly in Asia, and is one of the most common ingredients in both traditional cuisine and local medicine [5]. In recent years, this polyphenol has attracted increasing global interest due to its proven anti-inflammatory and antioxidant properties. Numerous studies have shown that curcumin may offer benefits in a variety of diseases of varying origins, such as cancer, arthritis, diabetes, hypercholesterolemia, cardiovascular disease, liver damage, ischemia, brain aging, neurodegenerative diseases, and retinal disorders [10,11,12,13]. Scientific interest in curcumin has also increased because it has been approved as a “generally safe” compound (GRAS) by the US FDA. WHO and EFSA state that it is safe to take up to 3 mg/kg of curcumin daily. Reported side effects are generally mild, but intake may interfere with some medications, altering their effectiveness, as in the case of antidepressants or drugs for cardiovascular disease [5]. This review aims at exploring the central hypothesis that curcumin, through its pleiotropic biological effects and interaction with the gut–adipose–brain axis, may represent a promising complementary strategy in the prevention and management of obesity-related metabolic and cognitive disorders.

## 2. Effects of Curcumin on Body Composition and Obesity: Efficacy, Challenges, and Strategies to Improve Bioavailability

Numerous randomized controlled trials have examined the effect of curcumin on body composition in cases of obesity. A 2021 meta-analysis highlighted that doses lower than 1000 mg per day did not produce significant effects, while doses of 1500 mg or more per day, taken for over a month, led to a significant reduction in BMI and other body parameters, especially when combined with lifestyle changes. However, the effectiveness of curcumin is limited by its poor water solubility, rapid metabolism, and high elimination rate, which reduce curcumin concentrations in the blood. This is further confirmed by studies in both mice and humans, where curcumin plasma concentrations remain very low even with high doses [7]. Fortunately, today several strategies have been developed to improve the bioavailability of curcumin in the body. Among the strategies to improve curcumin bioavailability, there are (1) co-administration with piperine, which reduces hepatic and intestinal metabolism [14]; (2) the use of phytosomes, where curcumin is bound to phospholipids, facilitating intestinal absorption; (3) encapsulation in nano-formulations (nanocurcumin) [7]; and (4) the use of curcumin in its amorphous form rather than crystalline form [15]. In general, it seems that a daily intake of 1500 mg of curcumin produces more promising results than daily doses of 1000 mg or less, particularly with regard to anthropometric measures in overweight or obese adults [16].

**Figure 2:**
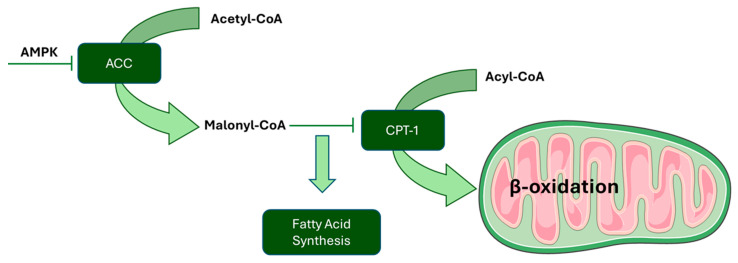
Effects of curcumin on metabolism regulation via AMPK. Curcumin modulates the activity of AMPK, activating catabolic processes and blocking anabolic ones. Specifically, it reduces lipogenesis, stimulates fatty acid oxidation, and prevents triglyceride synthesis by regulating specific enzymes through AMPK.

However, further studies with larger sample sizes, longer follow-up periods, and different administration methods are needed to evaluate the effect of curcumin on overweight or obese individuals. Several in vitro studies have examined the effects of curcumin on preadipocytes/adipocytes derived from murine 3T3-L1 fibroblasts. It was found that curcumin affects the viability rate and cell cycle regulation of preadipocytes. At high doses (about 30 µM), it promotes DNA fragmentation, activates caspases involved in apoptosis, and increases the apoptotic rate. At lower doses (<20 µM), however, it reduces cell proliferation without affecting viability. Preadipocyte differentiation is a complex process that includes mitotic clonal expansion (MCE) and terminal differentiation. During MCE, the retinoblastoma protein (Rb), a tumor suppressor protein that plays an important role in the initial step of adipogenesis, is phosphorylated, activating the transcription factor E2F and promoting cell cycle progression. Curcumin, at low doses, reduces Rb phosphorylation, slowing clonal expansion and the cell cycle, thus highlighting a potential antiadipogenic effect [16]. Curcumin affects the differentiation of preadipocytes. Regarding the terminal differentiation stage, the initial steps are similar for white and brown adipocytes and are regulated by transcriptional factors from the families of CCAAT enhancer binding proteins (C/EBPs, such as C/EBP α, β, and δ) and peroxisome proliferator-activated receptors (PPARs, especially PPARγ) [17]. When preadipocytes are exposed to inducers of differentiation, there is an initial and transient increase in C/EBPβ and δ, followed by an increase in C/EBPα and PPARγ, which are crucial for adipocyte gene expression. Alterations in these factors can significantly affect differentiation. Studies by MacDougald et al. showed that C/EBPα and PPARγ activity is regulated by Wnt-β catenin signaling, which acts as an inhibitory “switch” for these factors, blocking differentiation. When Wnt glycoprotein activates the Frizzled receptor, β-catenin is released and translocates into the nucleus, reducing the expression of C/EBPα and PPARγ and preventing differentiation. In this context, several studies have shown that curcumin can reduce white adipocyte differentiation by stimulating that pathway [16,18,19]. Curcumin induces metabolic changes through modulation of AMP-activated kinase (AMPK) activity. When AMP binds to AMPK, anabolic pathways are disrupted and catabolic pathways are activated. Specifically, activated AMPK: (1) reduces lipogenesis by inhibiting acetyl CoA carboxylase (ACC); (2) stimulates β-oxidation of fatty acids by increasing transport via carnitine palmitoyltransferase-1 (CPT-1); and (3) prevents triglyceride synthesis by inhibiting glycerol-3-phosphate acyltransferase-1 (GPAT-1). According to Ejaz et al., curcumin blocks anabolism and activates lipid catabolism through regulation of AMPK and associated enzymes (Figure 2) [20]. Data, also confirmed by J. Lone et al. [21], suggest that curcumin can promote white adipose tissue (WAT) browning. Specifically, administration of curcumin at doses below 20 µM in cultures of primary or 3T3-L1-derived adipocytes showed increased mitochondrial biogenesis (PGC-1α) and expression of markers typical of brown adipose tissue (BAT), such as the UCP1 protein, which dissociates the electron transport chain from ATP production, dissipating excess energy as heat. When adipocytes were treated with curcumin and dorsomorphine, an AMPK inhibitor, the increase in markers was blocked, whereas if curcumin was combined with AICAR, an AMPK activator, the effect was enhanced. These results indicate that curcumin may promote WAT browning, a process mediated by the AMPK pathway [21]. This process involves the transformation of preadipocytes or mature white adipocytes into multilocular beige adipose cells, which express genes related to thermogenesis. It occurs under physiological conditions and is an adaptive mechanism of WAT in adults, stimulated by cold, but also by the intake of particular foods and physical activity [7,21,22].

## 3. Adipocyte Remodeling, Systemic Inflammation, and the Role of Hypoxia in Obesity: Mechanisms and Implications

**Figure 3:**
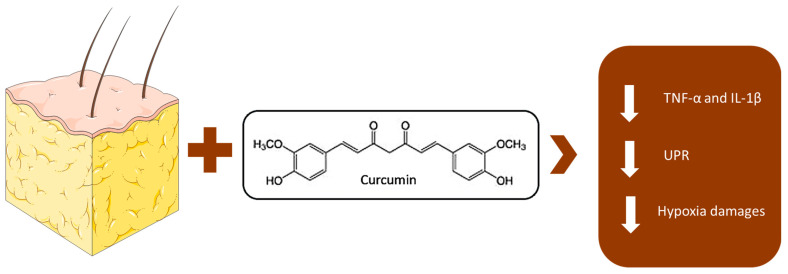
Curcumin, due to its antioxidant and anti-inflammatory properties, can reduce endoplasmic reticulum stress and inflammasome activation in hypertrophic adipocytes. Moreover, it modulates NF-κB activity and improves insulin sensitivity, helping to counteract the chronic inflammation associated with obesity.

When the energy balance changes, adipose tissue adapts by changing the number and size of adipocytes. This process, called “tissue remodeling”, is dysfunctional in obesity. Spalding et al. [23] observed that the number of adipocytes is established early in development, so those who are obese from childhood have a large number of adipocytes that remain unchanged, while those who develop obesity in adulthood have an increase in adipocyte size. In both cases, hypertrophic adipocytes in WAT are associated with systemic inflammation and insulin resistance [23]. Hypertrophic adipocytes cause inflammation in several ways. In particular, excessive endoplasmic reticulum stress activates the protein unfolding response (UPR), a process involving the sensor proteins IRE1, PERK, and ATF6, which are normally inhibited by a molecular chaperone. When misfolded proteins accumulate, as in hypertrophic adipocytes, these proteins are activated and stimulate inflammatory pathways, such as that of NF-KB. Studies by Kawasaki et al. (2012) showed that free fatty acids (FFA), abundant in the adipose tissue of obese people, activate the UPR response [24]. Specifically, treatment of 3T3-L1 adipocytes with FFA causes ROS accumulation, reticulum stress, and inflammation. Pretreatment with antioxidants reduces these effects. Researchers have also shown that decreasing reticulum stress, such as by molecular chaperones, may reduce inflammation, both in vitro and in vivo [24]. Hypertrophic adipocytes, under endoplasmic reticulum stress, release molecules that attract and activate macrophages. This phenomenon was observed by Kawasaki et al. [24] and confirmed by Furukawa et al. [25]. In one study, researchers incubated 3T3-L1 adipocytes with H_2_O_2_ for 24 h and observed an increase in the local production of pro-inflammatory cytokines, such as IL-6 and MCP-1, and a reduction in anti-inflammatory cytokines, such as adiponectin. This inflammatory effect can be counteracted by antioxidant treatments, such as *N*-acetylcysteine [25]. Hypertrophic adipocytes also influence the macrophage response through other mechanisms. Wang et al. [26] showed that the accumulation of ROS within adipocytes reduces the activity of phosphodiesterase 3 (PDE3), an enzyme that negatively regulates cAMP levels. This leads to abnormal PKA activation and increased lipolysis, resulting in increased basal FFA release. Thus, endoplasmic reticulum stress in adipocytes promotes the polarization of macrophages toward the M1 phenotype, both through the secretion of pro-inflammatory cytokines and by increased FFA release into the tissue [26]. Hypertrophic adipocytes show signs of necrosis, which could promote the formation of crown-like structures (CLSs), where macrophages aggregate to remove damaged tissue. Giordano et al. [27] suggest that chronic inflammation in CLS is caused by two main factors: (1) lipids released from dead adipocytes and (2) activation of pro-inflammatory cell death programs, such as pyroptosis, in degenerating adipocytes. This is supported by the observation that in hypertrophic adipocytes there is an increased expression of NLRP3 and caspase-1, indicators of inflammasome activation, which would be activated by cholesterol crystals in the cytoplasm of adipocytes [27]. Inflammasome activation in adipocytes can cause cell death and release of pro-inflammatory cytokines. However, some researchers, such as Cinti et al., found that in adipocytes, inflammasome activation by TNFα does not induce IL-1β release but stimulates lipocalin 2 secretion, which facilitates communication between adipocytes and macrophages, promoting pyroptosis and inflammation in neighboring macrophages [28]. Hypertrophic adipocytes can cause hypoxia in the surrounding tissue. In obesity, adipocyte growth in white adipose tissue (WAT) is not supported by adequate vasculature, reducing tissue oxygen levels and affecting cell metabolism. In response to this oxygen deficiency, cells activate the transcription factor hypoxia-inducible factor (HIF), composed of the subunits HIF-1β and HIF-1α. Under normoxic conditions, HIF-1α is degraded, but in hypoxia it is stabilized. Fujisaka et al. (2013) showed that hypoxia promotes inflammation in macrophages by inducing the expression of M1 genes, in a process that depends in part on HIF-1 factor [29]. What is even more interesting is that the same transcription factor is also activated in 3T3-L1 adipocytes [30]. Maintenance of a 24 h hypoxia regime in 3T3-L1 cells leads to several effects, including (1) increased levels of HIF-1α, (2) increased levels of TNF-α, IL-6, IL-1β, and INF-γ, (3) increased secretion of lactate and glycerol, a sign of glycolytic metabolism and lipolysis, (4) increased ROS, and (5) morphological and functional abnormalities in mitochondria. These results suggest that the relationship between adipocytes and hypoxia is complex and bidirectional: while proliferation and enlargement of adipose tissue can cause hypoxia, it is also true that exposure of adipocytes to low oxygen levels can induce metabolic changes typical of hypertrophic adipocytes [29]. Chronic low-grade inflammation, which develops in obesity, is a key factor in the pathogenesis of chronic degenerative diseases. Inflammation in obesity is triggered by the activation of the innate immune cells recruited to the adipose tissue. Dysfunctional adipocytes secrete inflammatory cytokines and chemokines, the adipocytokines, which recruit immune cells, amplifying the inflammatory response even at the systemic level [31]. This inflammatory state is reflected in the blood by a decrease in anti-inflammatory cytokines and adiponectin, and an increase in TNF-α, IL-1β, and leptin. Systemic inflammation is a major cause of insulin resistance, as it activates factors such as NF-κB and JNK kinase, which interfere with insulin signaling through phosphorylation of insulin receptor substrate-1 (IRS-1). In this context, approaches aimed at reducing inflammation, such as through nutraceuticals, could be an effective strategy to prevent or alleviate metabolic complications associated with obesity (Figure 3) [31,32,33].

## 4. Curcumin Anti-Inflammatory Effects in Obesity: Evidence from In Vitro, In Vivo, and Clinical Studies

There is a wealth of experimental evidence demonstrating that curcumin possesses potent anti-inflammatory activity, both in vitro and in vivo. In human THP1 monocytes, doses of curcumin ranging from 7.5 to 30 μM are able to counteract the effects of stimulation with LPS and INFγ by dose-dependently blocking the TLR4/MAPK and NF-κB pathways, as well as reversing M1 polarization [31,34]. Furthermore, also in THP1 macrophages, similar doses of curcumin (0–25.5 μM) reduce classical inflammasome activation, mediated by the transcriptional factor NF-κB and induced by treatment with phorbol myristate acetate (PMA) [35]. Much of the anti-inflammatory activity of curcumin on tissue macrophages appears to result from inhibition of the NF-κB signaling pathway. This anti-inflammatory effect has also been observed in adipocytes. For example, in 3T3-L1 adipocytes, a curcumin dose of 20 μM reduces the TNFα-induced pro-inflammatory effect by stabilizing the IκB inhibitor and limiting the translocation of NF-κB to the nucleus [36]. Similarly, in primary adipocytes of murine origin, a 10 μM dose of curcumin reduces pro-oxidant agent-induced endoplasmic reticulum stress by attenuating the UPR response and phosphorylation levels of the p65 subunit of NF-κB. Furthermore, in primary adipocytes, the same dose of curcumin (10 μM) counteracts the increased basal lipolysis by maintaining the inhibitory activity of PDE3 on PKA, even after stimulation with pro-oxidants. These results suggest that curcumin (20 μM) treatment may also be effective in counteracting inflammation caused by hypoxia. In one study, 3T3-L1 adipocytes were cultured for 24 h under conditions of low oxygenation (1% O_2_), similar to those found in adipose tissue of obese mice (P_O2_ = 7.6 mm Hg). This treatment caused several abnormalities, including activation of the inflammatory response, alterations in mitochondrial morphology, reduction in scavenger enzymes, increased ROS, lipid peroxidation, and protein oxidation. However, administration of curcumin (5–20 μM) was able to mitigate these cellular damages [26,30]. Some clinical trials have documented that curcumin treatment brings several benefits to markers of chronic systemic inflammation in overweight patients. For example, in one such trial, 84 overweight/obese patients, aged 25 to 50 years and suffering from nonalcoholic fatty liver disease (NAFLD), ingested curcumin capsules twice daily, after meals, for a total daily dose of 80 mg. After 3 months of treatment, a reduction in some serum pro-inflammatory markers, such as TNFα, high-sensitivity C-reactive protein (hs-CRP), and IL-6, was observed [31,37]. These results were further confirmed by a study involving 60 adolescent girls who were treated simultaneously with a light diet and curcumin (500 mg daily) for a period of 10 weeks. Similar to what was observed in the previous study, curcumin intake led to a significant reduction in serum levels of hs-CRP and IL-6 compared with the control group [38]. In another study, 50 patients with type 2 diabetes, aged 18–65 years, were supplemented with curcuminoids (300 mg daily) for 3 months. The treatment resulted in a significant reduction in insulin resistance parameters and circulating levels of FFA, which are associated with inflammation in obese patients [39]. Finally, a clinical trial showed that in obese patients with type 2 diabetes and depression, consuming curcuminoids (1500 mg daily) for one year led to a significant reduction in depressive symptoms. This effect could be attributed to decreased systemic inflammation (lowered levels of TNFα, IL-1β, and IL-6) and improved antioxidant response in the blood (increased glutathione peroxidase and superoxide dismutase), factors that could influence serotonin signaling at multiple levels [40]. Importantly, although some clinical trials have shown a reduction in inflammatory markers in obese patients treated with curcumin, there are also studies that have failed to achieve the same results [41]. In light of the findings, it is essential to conduct new clinical trials to conclusively confirm the anti-inflammatory effects of curcumin in humans as well. Table 1 reports the clinical studies.

## 5. Curcumin Role in Modulating Oxidative Stress and the Keap1-NRF2 Pathway in Obesity-Related Inflammation

**Figure 4:**
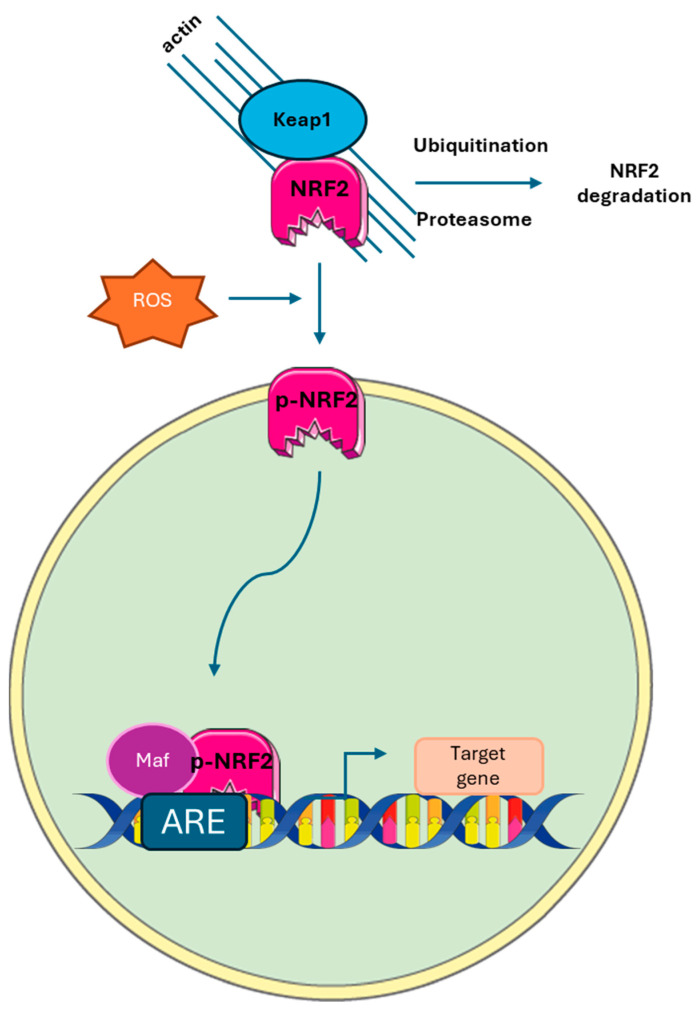
The Keap1-NRF2 pathway. Under normal conditions, Keap1 behaves as a negative regulator of NRF2, preventing its activation and thereby inhibiting its antioxidant response. Keap1 binds to NRF2, leading to its degradation and preventing the transcription of antioxidant genes. However, when the cell is exposed to certain stressors or stimuli, such as oxidative stress or toxins, Keap1 undergoes conformational changes or modifications. These alterations reduce Keap1 ability to bind NRF2, allowing NRF2 to stabilize and accumulate. Once released from Keap1, NRF2 translocates into the nucleus, where it binds to antioxidant response elements (ARE) in the DNA. This interaction activates the transcription of various genes involved in cellular defense mechanisms, including those that promote the production of antioxidant enzymes, detoxifying proteins, and other protective factors. Thus, the Keap1-NRF2 pathway plays a critical role in cellular responses to oxidative stress and maintaining cellular homeostasis.

Oxidative stress occurs when there is an imbalance between the production of oxidant molecules (such as oxygen and nitrogen radicals) and the ability of cells to neutralize them. This causes damage to cellular components such as lipids, DNA, and proteins. To counter these effects, the body has defense mechanisms, including activation of the Keap1-NRF2 pathway. Normally, Keap1 inhibits NRF2, preventing its antioxidant action. However, in the presence of certain stimuli, Keap1 is modified, releasing NRF2, which migrates into the nucleus and activates the genes responsible for the antioxidant response (Figure 4) [42]. Among these genes, there are those that code for enzymes such as glutamate-cysteine ligase, glutathione S-transferase, NADP(H) quinone oxidoreductase, and hemoxygenase 1. Some plant compounds, particularly polyphenols, can modulate this process [43]. A study in RAW 264.7 macrophages showed that curcumin, at doses below 40 µM, improved cellular resistance to hydroxyl radicals without being toxic. A dose of 5 µM, administered for 20 h, potentiated the response to oxidative stress, increasing cell viability and the activity of antioxidant enzymes. It also stimulated the translocation of NRF2 into the nucleus, activating the transcription of antioxidant genes such as hemoxygenase 1 (HO-1) [44,45,46]. Curcumin ability to modulate the NRF2-HO-1 pathway was also confirmed in renal epithelial cells and in mouse epidermal JB6 cells [46,47]. The mechanism by which curcumin promotes the NRF2 pathway is still debated. According to data by Shin et al., curcumin would be able to bind the Keap1 inhibitor, specifically cysteine 151, thus preventing polyubiquitination and subsequent degradation of NRF2 at the proteasome [47].

The ability of curcumin to activate NRF2, particularly in macrophages, is of great interest in obesity research, as oxidative stress plays a key role in inducing the inflammatory response associated with this condition [47].

## 6. The Role of Curcumin in Preventing Metabolic Diseases in Adolescence and Early Childhood

Metabolic diseases that develop during late adolescence, such as insulin resistance, type 2 diabetes, and NAFLD, tend to persist into adulthood. In this context, early and noninvasive interventions could be an effective solution to address eating disorders.

**Figure 5:**
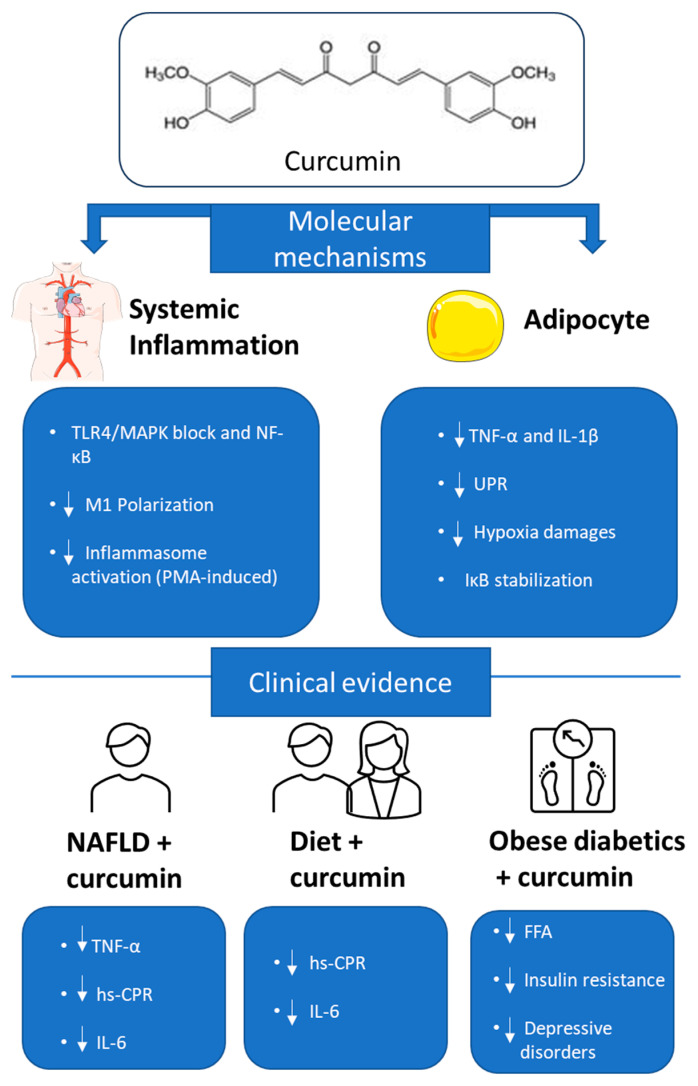
Anti-inflammatory effects of curcumin: molecular and clinical evidence. Image shows the main mechanisms by which curcumin exerts anti-inflammatory effects both systemically and in adipocytes. At the molecular level, curcumin blocks inflammatory signals mediated by TLR4/MAPK and NF-κB in macrophages, reduces M1 polarization and inflammasome activation. In adipocytes, it decreases TNF-α and IL-1β levels, reduces endoplasmic reticulum stress (UPR), limits hypoxia damage, and stabilizes IκB inhibitor. The bottom summarizes the clinical evidence: in patients with NAFLD, in combination with diet or in obese diabetic subjects, curcumin reduces inflammatory markers (TNF-α, IL-6, hs-CRP), free fatty acids (FFA), insulin resistance, and depressive symptoms.

A 2019 study of 60 adolescent girls with obesity found that taking 500 mg of 95% curcumin for 10 weeks improved metabolic as well as anthropometric parameters when combined with calorie control and physical activity monitoring. The analysis showed improvements in BMI, waist and hip circumference, HDL, and insulin levels. However, these benefits were not evident when compared with a placebo group [38]. Other studies suggest that early interventions, such as diets and exercise, have more lasting effects in children than in adolescents, supporting the idea that early nutritional interventions can improve long-term health [48]. The period from conception to 2 years of age, encompassing pregnancy, breastfeeding, and weaning, is considered “highly sensitive”, as adequate nutritional intake at this stage is critical to prevent the development of endocrine, metabolic, and immunological changes in adults, such as “metabolic syndrome”. Therefore, the research group of Du et al. studied the effects of curcumin in the early postnatal phase using an animal model of childhood obesity [49]. To simulate early overnutrition, “small litter” rats, which consume more milk and fat than “normal litter” rats, were used. After weaning, both groups were treated with curcumin or placebo for 10 days. The “small litter” rats showed metabolic changes, such as increased body weight, altered glucose tolerance, and changes in lipid levels, already observed in the pre-pubertal and pubertal stages. Three curcumin treatments were administered at different developmental stages: pre-pubertal (CUR13), pubertal (CUR16), and post-pubertal (CUR18). Results showed that CUR13, started before puberty and continued during puberty, had the greatest benefits, normalizing many parameters. In contrast, CUR18 had more limited effects. CUR13 and CUR16 treatments also positively influenced adipogenic pathways, suggesting that the pre-pubertal phase is crucial for preventing metabolic disorders and supporting the use of phytotherapeutics such as curcumin in children (Figure 5) [49].

## 7. Effects of Curcumin Treatment on Brain Inflammation Associated with Obesity

A 2012 study by Thaler et al. [50] suggests that brain and peripheral inflammation related to overeating are distinct phenomena with different origins. Peripheral inflammation develops slowly, over weeks or months, and is mainly caused by fat accumulation and changes in lipid metabolism. In contrast, brain inflammation occurs as early as a few days after a high-calorie diet, before there is a significant increase in body weight. According to the study, a dual phase of inflammation occurs in the brains of overfed mice and rats: an acute one in the first few weeks and a chronic one later. After three days of overeating, signals of NF-kB pathway activation and increased mRNAs of various inflammatory genes (IL-6, TNF-α, Socs3, CD86) and GFAP (astrocyte marker) are observed in the hypothalamus, which collectively underscore the early recruitment of astrocytes and microglia to the hypothalamic site [50].

Initial inflammatory events appear to play a protective role for the brain, as they are accompanied by increased expression of the chaperone protein Hsp72 and tend to resolve over time, returning the hypothalamus to a normal state. However, if the hypercaloric diet persists for a long time, inflammation and gliosis become permanent, with irreversible damage to neurons. In animals hyperfed for eight months, cells in the arcuate nucleus undergo degeneration, and the loss of these neurons is sufficient to cause excessive weight gain. In addition, astrogliotic phenomena have also been observed in obese patients, with a correlation between the severity of astrogliosis and BMI, but without relation to age or sex [50]. In 2017, the group of Valdearcos et al. [51] clarified the role of microglia in hypothalamic inflammation. The researchers found that: (1) both microglia and astrocytes accumulate in the hypothalamus of mice fed a diet rich in saturated fatty acids (SFA); (2) after only 24 h of exposure to palmitic acid in a dish, microglia produce significantly higher amounts of IL-6, TNFα, and MCP-1 than astrocytes [51]. Overall, these results suggest that microglia play a key role in coordinating the interaction between pro-inflammatory cells in the hypothalamus.

Given the action of curcumin on peripheral macrophages, it is not surprising that this nutraceutical also affects central microglia, modulating its polarization. A study by Zhang et al. [52] examined the effects of curcumin pretreatment for 2 h on BV2 cells, followed by LPS stimulation for 24 h. Curcumin doses lower than 10 µM prevented M1 polarization without causing toxicity, reducing pro-inflammatory markers (iNOS, IL-1β, IL-6) and increasing anti-inflammatory markers (arginase 1, IL-4), as shown by qPCR and ELISA. M1 to M2 polarization was confirmed by a decrease in CD16/32+ cells and an increase in CD206+ cells detected by flow cytometry. Furthermore, protein analysis of cell extracts revealed: (1) a decrease in TLR4 expression and phosphorylation levels of NF-kB pathway proteins (p-IKB and p-NFKB); (2) an increase in TREM2 receptor, a microglia-specific anti-inflammatory protein that antagonizes TLR4 activation [52]. In summary, these data suggest that curcumin may influence microglia polarization by acting on the interaction between TLR4 and TREM2. Its anti-inflammatory role has been further confirmed by other studies. In one of these, mice fed a hypercaloric diet containing 30% sucrose for 56 days showed a significant increase in markers of neuroinflammation and microglial activation in the hippocampus and hypothalamus, starting from the 14th day of overfeeding. This demonstrates that brain inflammation induced by a hypercaloric diet is an early phenomenon compared to systemic inflammation. Curcumin treatment, starting from doses of 20 mg/kg, was able to: (1) significantly reduce pro-inflammatory markers in the brain; (2) normalize TLR4 expression levels and NF-kB phosphorylation; (3) stimulate neurogenesis in the dentate gyrus; and (4) protect the brain from damage caused by chronic exposure to fructose [53].

## 8. Impact of Curcumin on Gut Microbiota and Metabolism: Implications for Obesity and Health

The human microbiota is mainly composed of bacteria (about 50 different phyla), but also includes viruses, fungi, and other species, forming an extremely large and diverse community of microorganisms. In fact, the genome of the microbiota, known as the microbiome, is about 150 times larger than the human genome [5]. In particular, among the most studied microorganisms are bacteria, which play a fundamental role, colonizing the intestine in much higher quantities than other body surfaces. In fact, it has been documented that their density in the colon can reach approximately 10^11^–10^12^ cells per millimeter [54].

Intestinal bacterial colonization is influenced by many factors, including age, diet, exercise, and the state of health of the subject. In general, these bacteria establish a symbiotic relationship with the human organism, feeding on the nutrients present in the intestine and, in exchange, performing crucial functions for the protection, metabolism, and structure of the intestine [3,55]. For example, intestinal bacteria: (1) produce B and K vitamins; (2) participate in the metabolism of lipids and proteins; (3) promote the fermentation of complex carbohydrates (such as dietary fibers), contributing to the synthesis of short-chain fatty acids (SCFAs), molecules with important anti-inflammatory, anti-tumor, and energy-providing properties for colon intestinal cells; (4) preserve the integrity of the intestinal barrier; and (5) modify the intestinal microenvironment to prevent colonization by pathogens. For this reason, it is not surprising that there is a correlation between dysbiosis (altered composition of the intestinal microbiota) and several pathologies. This applies not only to diseases of the gastrointestinal tract, such as irritable bowel syndrome, colorectal cancer, and celiac disease, but also to extra-intestinal conditions, such as obesity, diabetes, asthma, allergies, and some neurodegenerative diseases [3,5,54,55].

Several studies have shown that oral administration of curcumin can influence the composition of the intestinal microbiota. For example, the group of Shen et al. [56] treated mice for 15 days with a mixture of curcumin (40.9%), demethoxycurcumin (33.2%), and bidemethoxycurcumin (23.3%) at a dose of 100 mg/kg to observe the effects on the microbiota. The analysis of bacterial families present in the intestine revealed a modulation of groups such as Prevotellaceae, Bacteroidaceae, and Rikenellaceae, particularly in the genera Bacteroides and Alistipes [56]. Similarly, Li et al. [57] showed that the inclusion of 0.2% curcumin in food for 10 weeks reduced insulin resistance and hepatic fat accumulation and altered the microbiota in a mouse model of NAFLD. In particular, curcumin affected the expression of the genera Bacteroides, Parabacteroides, Alistipes, and Alloprevotella, which are responsible for the increase in SCFA in the caecum and colon [57]. Furthermore, another research group confirmed that curcumin treatment at a dose of 200 mg/kg for 14 weeks resulted in metabolic improvements in mice with NAFLD, as well as modulating the levels of Dorea and Butyricicoccus, bacteria associated with obesity [58].

Primary bile acids are metabolites produced by the liver from cholesterol, such as cholic acid and chenodeoxycholic acid. After being conjugated with glycine or taurine, they are secreted and stored in the gallbladder until digestion begins. Once released into the intestine, these acids are metabolized by intestinal bacteria into secondary bile acids, such as deoxycholic acid and lithocholic acid, which are then reabsorbed and released into the bloodstream, influencing various organs and tissues. At the adipose tissue level, secondary bile acids activate the TGR5 receptor, protein kinase A (PKA), and the expression of the UCP1 protein, stimulating thermogenesis in BAT and the “browning” process in WAT. Therefore, the regulation of this mechanism could represent an effective strategy for body weight control. It has been demonstrated that several polyphenols exert their anti-obesogenic action precisely by modifying the intestinal microbiota and the metabolism of bile acids [59]. Among the most studied polyphenols, resveratrol and curcumin stand out. For example, a study by Han et al. [60] showed that in hypernourished mice, treatment with curcumin (100 mg/kg per day) for 6 weeks reduced weight gain and improved cold tolerance. Fecal microbiota transplantation (FMT) experiments demonstrated that the thermogenic effect induced by curcumin, mediated by UCP1 activation, directly depended on the intestinal microbiome. Furthermore, the results revealed that: (1) the increase in adaptive thermogenesis in mice treated with curcumin was accompanied by an increase in circulating levels of deoxycholic acid (DCA) and lithocholic acid (LCA); (2) deletion of the TGR5 receptor abolished the thermogenic effect in brown adipose tissue induced by curcumin. In summary, these data suggest that a significant portion of the anti-obesogenic activity of curcumin is related to the modulation of bile acid metabolism [60].

## 9. Curcumin Promotes the Well-Being of the Intestinal Barrier

The intestinal wall has several defense mechanisms against microbial invasion: (1) intestinal alkaline phosphatase (IPA), an enzyme produced by enterocytes that neutralizes bacterial endotoxin (LPS); (2) a mucin layer that acts as a barrier between bacteria and the intestinal epithelium; (3) tight junctions that create a natural barrier against the diffusion of LPS into the bloodstream; and (4) antibacterial proteins secreted by Paneth cells, released into the intestinal lumen. However, maintaining intestinal health also depends on the individual’s dietary habits [61]. In this regard, it is widely documented that prolonged consumption of an unbalanced diet, such as the Western diet, causes dysbiosis of the bacterial flora, which results in: (1) the proliferation of pyrogenic Gram-negative bacteria; (2) increased intestinal permeability; (3) the passage of LPS into the systemic circulation; and (4) the exacerbation of the condition of chronic inflammation typical of subjects suffering from obesity and other associated metabolic diseases [3].

In a study by Ghosh et al., [62] mice lacking the LDL receptor (LDLR-/-) were fed a high-fat, high-cholesterol diet for 16 weeks. The results showed a significant increase in serum LPS levels and a reduction in intestinal alkaline phosphatase activity. However, administration of curcumin (100 mg/kg/day) was able to restore alkaline phosphatase activity and reduce intestinal permeability. Next, the researchers investigated the mechanisms underlying this effect of curcumin. They treated CaCo-2 cells with curcumin (5 μM) for 48 h and then exposed them to LPS overnight, simulating intestinal inflammation typical of obesity. The results showed that curcumin was able to restore the levels of claudin 1 and zonula occludens 1 (ZO-1) in tight junctions, suggesting a mechanism of modulation of intestinal permeability [62]. These results were further confirmed by a subsequent study by the same research group. In this case, human intestinal CaCo-2 cells were pretreated with curcumin (5 μM) for 48 h and subsequently exposed to LPS for 24 h. Immunocytochemical analyses showed that curcumin reduced the loss of expression of actin, ZO-1, and claudins 1 and 7 in tight junctions. This protective effect was due to the anti-inflammatory properties of curcumin. In particular: (1) curcumin decreased the secretion of IL-1β produced by macrophages and intestinal epithelial cells; (2) suppression of IL-1β signaling caused the inhibition of p38 MAPK and myosin light chain kinase (MLCK) in intestinal epithelial cells; (3) this prevented the phosphorylation of proteins involved in tight junctions; (4) the final outcome was the reduction in intestinal permeability. In summary, these data suggest that curcumin plays a fundamental role in maintaining the health of the intestinal barrier, promoting the activity of alkaline phosphatase, and reducing the secretion of pro-inflammatory cytokines that could compromise intestinal permeability and trigger systemic inflammation [61]. Gan et al. (2019) [63] studied the effects of chronic curcumin treatment on pigs, animals that, after weaning, experience intestinal dysbiosis and inflammation. In this study, curcumin administration led to a significant reduction in *Escherichia coli* proliferation and decreased the expression of TLR4, IL-1β, and TNF-α in the intestine [63].

Similarly, in a mouse model of type 2 diabetes, curcumin treatment at a dose of 200 mg/kg for 10 weeks produced the following effects: (1) modified the composition of the gut microbiota; (2) reduced the expression levels of TLR4 and phosphorylation of NF-kB in the intestine; (3) promoted the increase in occludin and ZO-1 levels in tight junctions; and (4) improved insulin resistance [64].

A 2023 study confirmed that curcumin can attenuate the intestinal NF-kB pathway in a high-fat diet (HFD) animal model. In this study, treatment with a hypercaloric diet for 8 weeks, followed by co-treatment with curcumin (107 mg/kg/day) for the last 4 weeks, significantly counteracted the increase in TLR4, NF-kB, and IL-1β expression in the colon of animals subjected to the hypernourished diet. These results suggest that curcumin plays a fundamental role in maintaining the integrity of the intestinal barrier, and among the mechanisms involved, there is the modulation of the TLR4/MyD88/NF-kB pathway [65].

## 10. Curcumin and Gut Health: Potential Neuroprotective Effects Through the Gut–Brain Axis in Neurodegenerative Diseases and Obesity

Recent studies suggest that improving gut health may help to reduce neuroinflammation and neuronal degeneration. In particular, it has been shown that curcumin administration (60 mg/kg) for 49 days brought several benefits in a mouse model of Parkinson’s disease, induced by the administration of the toxin MPTP in the substantia nigra. Specifically, curcumin reduced damage to dopaminergic neurons, improved motor deficits, and, at the same time, increased the expression of the proteins occludin and ZO-1 in the tight junctions of the intestinal epithelium. Furthermore, it modulated the intestinal bacterial flora and reduced inflammation at the intestinal level [66,67]. Similar results were also obtained in the same model by fecal transplantation [68]. Furthermore, Lamichhane et al. demonstrated that curcumin treatment (4 g/kg) for 14 weeks in 3XTg-AD mice, an experimental model of Alzheimer’s disease, helped to reduce spatial memory deficits and mitigate intestinal dysbiosis exacerbated by high-calorie diets [69]. In summary, these results suggest a positive connection between brain health and gut health. This link appears to be present even in obesity, as evidenced by the fact that healthy mice, receiving microbiota from obese donors, develop not only cognitive problems and brain inflammation but also dysbiosis and increased intestinal permeability [70].

In contrast, obese mice transplanted with microbiota from healthy donors show an improvement in cognitive deficits and a reduction in markers of brain inflammation [71].

Furthermore, even in conditions of obesity, the use of plant compounds can exert neuroprotective effects, positively influencing the gut–brain axis. This was highlighted by a study conducted by Syeda et al. [72], in which mice previously fed a hypercaloric diet for 3 months were co-treated with a mix of bioactive foods. These included: (1) curcumin; (2) nopal, a Mexican plant rich in polyphenols; (3) chia seeds, a source of linolenic acid and *n*-3 fatty acids; and (4) soy proteins, which promote the well-being of the intestinal flora. The results show that treatment with these bioactive foods led to a reduction in body weight, an increase in lean mass, and a decrease in fat mass. Furthermore, at the intestinal level, the microbiota was modulated, the expression of TLR4 and TNFα was reduced, and the expression of occludin in tight junctions was increased. At the brain level, the treatment induced an increase in dendritic spines in the prefrontal cortex and improved the animals’ cognitive performance in tests of working memory and object recognition [72].

These data suggest that dietary correction can have positive effects on the brain even in the presence of obesity, acting directly on the gut–brain axis. However, further research is needed to understand the specific role of curcumin in this process.

## 11. Conclusions

In conclusion, growing evidence suggests a significant link between gut health, neuroinflammation, and metabolism, with important implications for the treatment of obesity and neurodegenerative diseases. The gut microbiota, in fact, plays a crucial role in influencing intestinal permeability and activating inflammatory pathways that, in turn, can influence brain health. Several studies have shown that the modulation of microbiota, through the use of bioactive compounds such as curcumin, can exert protective effects at both the gut and brain levels, reducing inflammation, improving cognitive function, and positively influencing body composition. Curcumin and other plant polyphenols appear to act through mechanisms involving the regulation of intestinal receptors, such as TLR4, and the modulation of the intestinal barrier, suggesting that treatment with these compounds could represent a promising therapeutic strategy to counteract gut–brain axis dysfunctions, particularly in conditions of obesity.

However, despite the encouraging results from preclinical studies, the translational potential of curcumin in clinical practice remains to be fully validated. The current clinical evidence is still limited, often characterized by small and heterogeneous study populations, short-duration treatments, low bioavailability of the compound, and considerable interindividual variability in response. These limitations underscore the need for rigorous, large-scale, placebo-controlled clinical trials using standardized and optimized curcumin formulations to truly assess its efficacy and safety in humans. In particular, future research should aim at defining the specific microbial targets and signaling pathways modulated by curcumin and its metabolites, as well as clarifying the extent to which these changes contribute to systemic metabolic and neurological improvements. Additionally, the development of novel delivery systems, such as nano-encapsulation, liposomal formulations, or microbiota-directed vectors, may significantly enhance its bioavailability, stability, and targeted activity, improving therapeutic outcomes. Moreover, integrating omics-based technologies (e.g., metagenomics, metabolomics, transcriptomics) will be essential to unravel individual variability in response to curcumin supplementation and to identify potential biomarkers of efficacy and responsiveness. This approach could ultimately support the implementation of personalized nutritional and therapeutic strategies, tailored to the patient’s microbiota composition, genetic background, and metabolic profile. In this context, curcumin may emerge not only as a nutraceutical of interest but also as a potential adjuvant in precision medicine, especially in the management of obesity and neuroinflammation-related disorders. Nevertheless, a deeper mechanistic understanding and robust clinical validation are essential prerequisites before curcumin can be fully integrated into evidence-based therapeutic protocols.

## Figures and Tables

**Table 1:** Clinical studies with curcumin.

Patients	Inclusion Criteria	Experimental Group Composition	Treatment	Main Effects	Type of Study	Reference
84 overweight/obese patients suffering from NAFLD, male and female	25–50 years old, NAFLD diagnosed using ultrasonography and 25 ≤ BMI < 35 kg/m^2^.	23 male+ 19 females in each group	Nano-curcumin capsules, twice daily, 80 mg/day, for 3 months	-decreased fatty liver degree -improvement in glycaemic, lipid and insulin resistance parameters (fasting glucose, HbA1c, HOMA-IR and others) -reduction of inflammatory response (TNFα, hs-CRP, IL-6 reduction)	double-blind, randomized, placebo-controlled clinical trial	[37]
60 overweight or obese adolescent girls	13–18 years old, having a menstruation cycle more than 6 months. overweight and obesity were defined as body mass index (BMI) percentile for age between 85th and 95th and BMI percentile for age more than 95th, respectively.	30 subjects in each group	-slight weight loss diet -500-mg curcumin capsule a day for 10 weeks.	-reduction of inflammatory markers (hs-CRP, IL-6) -increased antioxidant response	double-blind. randomized placebo-controlled clinical trial	[38]
100 patients with T2DM, male and female (50/experimental group palcebo/curcumin)	18–65 years old. BMI ≥ 24.0; fasting blood glucose ≥ 7.0 mmol/L or postprandial blood glucose ≥ 11.1 mmol/L)	25 male+ 25 females in placebo group 24 male+ 26 females in treated group	-150 mg curcuminoids capsule twice daily, 300 mg daily, for 3 months	-improvement of diabetic condition and reduction of fasting glucose, HbA1c, and HOMA-IR -decreased concentration of serum total FFAs	randomized, double-blind, placebo- controlled trial	[39]
227 patients with T2DM and depression, male and female	>35 years old. BMI ≥ 23 kg/m^2^ and well-controlled blood glucose (glycated hemoglobin [HbA1c] < 6.5% and fasting plasma glucose [FPG] < 110 mg/dL).	54 male+ 80 females in placebo group 62 male+ 73 females in treated group	-curcuminoids 3 capsules, twice daily, (1500 mg daily) for one year	-improved depression severity and plasmatic serotonin levels -improvement of glycaemic and insulin resistance indices (HbA1c, HOMA-IR) -reduction of inflammatory markers (TNFα, IL-1β, IL 6) -increased antioxidant defence (GPX and SOD)	randomized, double-blind, placebo- controlled trial	[40]

## Abbreviations

ACC	acetyl CoA carboxylase
AMPK	AMP-activated kinase
BAT	brown adipose tissue
CLSs	crown-like structures
CPT-1	carnitine palmitoyltransferase-1
DCA	deoxycholic acid
FFA	free fatty acids
FMT	Fecal microbiota transplantation
GPAT-1	glycerol-3-phosphate acyltransferase-1
HFD	high-fat diet
HIF	Hypoxia-inducible factor
HO-1	hemoxygenase 1
hs-CRP	high-sensitivity C-reactive protein
IPA	intestinal alkaline phosphatase
LCA	lithocholic acid
MCE	mitotic clonal expansion
MLCK	myosin light chain kinase
NAFLD	nonalcoholic fatty liver disease
PDE3	phosphodiesterase 3
PKA	protein kinase A
PMA	phorbol myristate acetate
PPARs	peroxisome proliferator-activated receptors
Rb	Retinoblastoma protein
SFA	saturated fatty acids
SCFA	short-chain fatty acids
UPR	protein unfolding response
WAT	white adipose tissue
ZO-1	zonula occludens 1
